# A Finite Element Analysis of a New Dental Implant Design: The Influence of the Diameter, Length, and Material of an Implant on Its Biomechanical Behavior

**DOI:** 10.3390/ma18122692

**Published:** 2025-06-07

**Authors:** Pedro González-Mederos, Jennifer Rodríguez-Guerra, Jesús E. González, Alberto Picardo, Yadir Torres

**Affiliations:** 1 Departamento de Biomateriales Cerámicos y Metálicos, Centro de Biomateriales, Universidad de La Habana, Ave. Universidad s/n Entre G y Ronda, Vedado, La Habana 10400, Cuba; pedroernestog97@gmail.com (P.G.-M.); jennifer.rodriguez.guerra98@gmail.com (J.R.-G.); 2 Grupo de Biomecánica, Facultad de Mecánica, Universidad Tecnológica de la Habana “José Antonio Echeverría”, Dirección Calle 114, # 11901, e/Ciclovía y Rotonda, Marianao, Cujae, La Habana 19390, Cuba; 3 Departamento de Ingeniería del Diseño, Escuela Politécnica Superior de Sevilla, Universidad de Sevilla, Calle Virgen de África, 7, 41011 Sevilla, Spain; apicardo1@us.es; 4 Ingeniería y Ciencia de los Materiales y del Transporte, Escuela Politécnica Superior de Sevilla, Universidad de Sevilla, Calle Virgen de África, 7, 41011 Sevilla, Spain

**Keywords:** dental implant, finite element analysis, biomechanical behavior, stress shielding Phenomenon, β-Ti alloy, implant dimensions

## Abstract

It is widely recognized that excessive stress and/or strain can lead to peri-implant bone atrophy; therefore, the clinical success of dental implants is intrinsically related to their biomechanical behavior. This study evaluates the influence of the diameter, length, and material [Ti6Al4V (α+β Ti) and Ti35Nb7Zr5Ta (β-Ti)] of a novel cylindrical dental implant on stress and strain levels within maxillary bone of type II quality. The implant design aims to ensure an appropriate distribution of stresses and strains within the peri-implant bone structures (cortical and trabecular bones) while also facilitating surgical machining by requiring a simple, linear, and less expensive bone incision. This approach minimizes the risk of thermal necrosis, a common complication in osteotomies for conical implants that can lead to peri-implant bone loss. Using finite element analysis, stress and strain patterns were evaluated in the maxillary second premolar region under static delayed loading. The results reveal that the cortical bone strains remained below the critical threshold (0.003) to prevent resorption. In the trabecular bone, only larger diameter/length configurations satisfied the previous strain criterion. In all simulations, trabecular bone stress remained below 3 MPa, whereas cortical bone stress peaked at 78 MPa. Notably, the implant model with the largest diameter/length minimized stress and strain concentrations in type II bone when compared to smaller designs, thereby demonstrating its biomechanical advantage.

## 1. Introduction

In recent decades, substantial progress has been achieved in prosthetic dentistry, leading to notable improvements in both dental implant technology and surgical techniques. The primary focus of these advances has been to ensure predictable clinical outcomes while simultaneously enhancing functional performance and esthetic results in patients with complete or partial edentulism [1]. The efficacy of prosthetic rehabilitation depends on multiple variables that can influence the biomechanical interaction between the implant and the osseous tissue [2,3]. These factors encompass the exact positioning of the implant, the inherent mechanical and structural properties of the bone tissue, the mechanical and geometric features of the implant itself, and the intensity and type of load transmitted from the implant to the surrounding bone [4,5,6]. Additionally, it is necessary to consider patient-specific factors such as smoking habits and bacterial environment [7,8,9].

The success of dental implant treatment depends, among other factors, on the efficient transfer of occlusal loads at the bone–implant interface [10]. Multiple elements, including the loading regimen, implant surface topography, available bone volume, as well as the material properties and design characteristics of the implant, influence this load transfer [11]. An optimal design can mitigate stress and strain concentrations while improving their distribution patterns, thus improving the probability of long-term implant survival [12]. Stress and strain distributions in the peri-implant bone are directly affected by the nature of the applied load [13]. Excessive mechanical loading (overload conditions) may initiate bone micro-fractures, potentially leading to implant loosening or catastrophic failure. Furthermore, overload situations can cause accelerated bone resorption in the peri-implant region and reduce trabecular bone density [14]. Regarding implant materials, they must demonstrate essential characteristics including biocompatibility, structural durability, and superior resistance to corrosion, wear, and mechanical fracture [15].

The selection of an appropriate dental implant necessitates a comprehensive evaluation of the residual alveolar bone, incorporating an assessment of the vertical bone height and mesiodistal dimensions of the edentulous space, to achieve optimal biomechanical performance and esthetic results [16,17]. Clinical guidelines recommend maintaining a minimum distance of 1.25 mm between an implant and adjacent natural teeth, providing adequate space to support bone and periodontal ligament tissue while ensuring sufficient vascular supply for successful osseointegration. Additionally, a minimum circumferential bone thickness of 0.5 mm surrounding the implant must be preserved to ensure the long-term clinical stability and success of prosthetic rehabilitation [18,19]. Post-extraction bone resorption in the maxilla leads to reduced residual bone height, particularly in the posterior region, where proximity to the maxillary sinus complicates the placement of standard-length implants [20]. Although bone grafting and sinus lift procedures are well-established treatment options, they are associated with increased morbidity and a prolonged duration of treatment. Short implants provide a minimally invasive solution without compromising primary stability as larger diameters and optimized implant body geometry can compensate for reduced length, thereby enhancing initial retention in low-density maxillary bone [21].

It is important to note that bone tissue responds to alterations in the loading conditions to which it is subjected. This phenomenon, known as bone remodeling, involves the ability of the bone to adapt and modify itself to achieve a balance between strength and resistance [22,23,24]. However, overload conditions can lead to bone fracture, fatigue failure, and detrimental consequences, including marginal bone loss or even osseointegration failure [25,26]. Peri-implant bone resorption can be triggered by various factors, such as surgical trauma, bacterial infections, and states of overload or underload, but to a lesser extent [6,27,28]. Overload in the peri-implant bone can occur due to deficiencies in the load transfer mechanisms, such as malocclusion, incorrect use of the implant, incorrect design of the prosthetic crown and/or implant, and improper placement of the implant. Consequently, this can result in high stress concentrations and/or strains at the bone–implant interface and, ultimately, bone resorption [29,30].

In essence, biomechanical load transfer at the bone–implant interface constitutes a critical determinant of dental implant success. Optimal implant design, including its geometry, diameter, length, and three-dimensional positioning within the maxilla, plays a pivotal role in governing the occlusal load distribution and the subsequent bone adaptive response [31,32,33]. A design that promotes a balanced load distribution and minimizes stress concentrations can help prevent bone resorption and improve the durability and clinical effectiveness of implant rehabilitations [34,35,36].

The use of finite element analysis (FEA) in prosthetic dentistry has become a predominant quantitative method to investigate the biomechanical behavior of dental implants in various clinical scenarios [37,38]. This technique enables the prediction of stress and strain distributions in peri-implant regions while considering multiple variables, including implant and prosthesis design, load magnitude and direction, bone mechanical properties, and other case-specific conditions [39]. The principal advantage of the FEA method lies in its ability to simulate the complexity of real clinical situations. Enabling the modeling of complex geometries and materials and the interactions between various components of the biomechanical system facilitates a more accurate understanding of the mechanical behavior within bone [40,41]. Consequently, this approach allows for the identification of potential overloaded regions or underloaded zones that may trigger bone resorption or implant failure [42,43,44]. However, it must be recognized that FEA studies are not exempt from certain assumptions and limitations. These encompass the selection of material properties, boundary condition definitions, interface characterizations between components, and the general modeling methodology [35,45]. The validity of FEA-derived results depends on the rigor of these assumptions and considerations and on the precise experimental validation of the models. Furthermore, these results are used to optimize the design of dental implants by incorporating modifications in geometry and dimensions [46,47]. In summary, using FEA for examining dental implant biomechanics offers significant benefits by enabling comprehensive simulation and analysis of complex bone–implant system interactions [48]. Nevertheless, the results must be interpreted cautiously as they remain subject to the inherent assumptions and limitations of the modeling approach, as previously outlined [49].

Ti6Al4V (α+β Ti) alloys are widely used in implant applications. However, their high elastic modulus (~110 GPa) and limited bioactivity can lead to stress concentrations at the bone–implant interface and contribute to peri-implant bone resorption. In contrast, β-phase titanium alloys such as Ti35Nb7Zr5Ta (β-Ti) present significant advantages, including a lower elastic modulus (~55 GPa), more similar to cortical bone (~10–30 GPa), along with improved corrosion resistance and biocompatibility [50]. Recent computational and in vivo studies have shown that β-Ti alloys promote more favorable strain distributions, reducing stress shielding and improving load transfer at the bone–implant interface. However, comprehensive evaluations of their micromechanical behavior remain limited [51].

Although widely used, conical implants exhibit critical biomechanical and surgical limitations: (1) their design induces stress concentration at the apex, increasing the risk of microfractures in high-density bone (types I and II); (2) complex bone preparation (tapered surgical drilling) may cause overheating and necrosis, leading to cortical bone loss, and (3) reduced initial bone-to-implant contact at the apical portion compromises early osseointegration [52]. Cylindrical implants offer key advantages as a potential solution: (a) uniform load distribution by eliminating localized stress points; (b) simplified (straight) and less invasive osteotomy, preserving bone integrity and minimizing thermal risks; and (c) greater initial bone contact area, enhancing osseointegration in high-density bone (type II) [53]. The standardized drilling protocol for cylindrical implants reduces surgical time, instrument costs, and thermal complications compared to conical systems, offering significant economic advantages in high-density bone [54]. This perspective challenges the approach paradigm, suggesting that cylindrical designs optimize both biomechanical performance and cost-efficiency in specific bone phenotypes, with direct implications for clinical planning and long-term implant longevity. A novel single-component cylindrical dental implant was designed, offering significant advantages: it eliminates the microgap and connection interfaces present in two-piece systems, which are prone to bacterial colonization, screw loosening, and galvanic corrosion. In addition, it improves biomechanical stability by distributing occlusal forces uniformly through a single-unit structure, reducing stress concentration at prosthetic junctions, and it simplifies clinical workflows by avoiding abutment seating inaccuracies, thereby improving primary stability, a key requirement for early functional loading.

The objective of this study is to assess the impact of the diameter, length, and material (Ti6Al4V (α+β Ti) and Ti35Nb7Zr5Ta (β-Ti)) on the biomechanical behavior of a new cylindrical dental implant model. Specifically, its influence was evaluated on the maximum levels of von Mises equivalent stresses and von Mises strains in the peri-implant bone (cortical and trabecular bone) of the second premolar region of the maxillary.

## 2. Materials and Methods

### 2.1. Dental Implant Models

**Figure 1:**
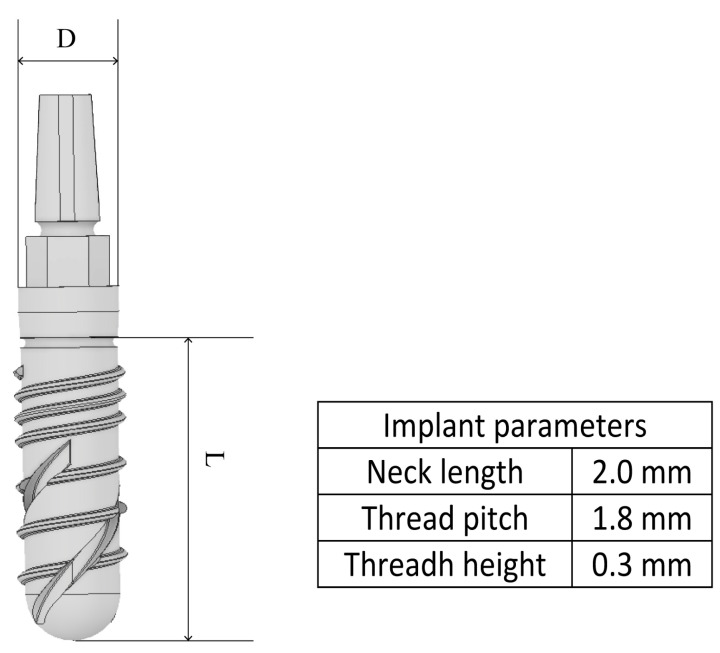
The cylindrical implant design used in the simulations and its main parameters.

The geometry of a single-component dental implant model used in previous studies was modified to reduce production costs and simplify its anchoring process in the maxilla [55,56]. Specifically, the taper of the referenced design was significantly reduced. Based on these modifications, six variants of the new single-component dental implant model (Figure 1) were obtained in the Autodesk Inventor 2020 software (Autodesk Inc., San Francisco, CA, USA), differentiated by their length and diameter values (Table 1). The implants had a thread that extended throughout the length of the implant body to which a second thread was added in the proximal area (close to its neck), both with a rectangular profile. In the implant body, two helical grooves were extended to enable self-tapping into the maxillary bone. Furthermore, to determine the influence of its dimensions on its biomechanical behavior, the length of the threaded portion (implant body, L) and the diameter of its neck (D) were varied (Table 1). 

### 2.2. The Assembly of the Crown Dental Implant System

To place the implants, the maxilla model obtained by Pérez from medical image processing was used [57]. The maxilla model, including adjacent teeth to the premolar, was modeled as a single solid body. Cuts were made to the model, and a geometry smoothing process was carried out at the limits of the premolar area to facilitate processing in the simulation software.

In Autodesk Inventor software, the six dental implant models were assembled with a ceramic crown, corresponding to the second premolar. Then, threaded holes were designed in the jaw according to the dimensions of each dental implant, and the crown dental implant systems were placed in these to form four assemblies. Subsequently, the assemblies were exported in .sat format to the Abaqus/CAE simulation software (6.13).

### 2.3. Analysis Using the Finite Element Method

The von Mises equivalent stress (VMES) and von Mises strain values (VMS) in the cortical and trabecular bones were obtained by the FEA using the Abaqus/CAE simulation software (Simulia Corp, Vélizy-Villacoublay, France, version 6.13). The system components were exported to Abaqus as separate parts, where material definitions were established and the mechanical properties specified in Table 2 were assigned. Cortical and trabecular bone (type II quality) were modeled with anisotropic material properties, while isotropic properties were assigned to the implant and crown. Additionally, all materials were treated as homogeneous volumes exhibiting linear elastic behavior.

**Figure 2:**
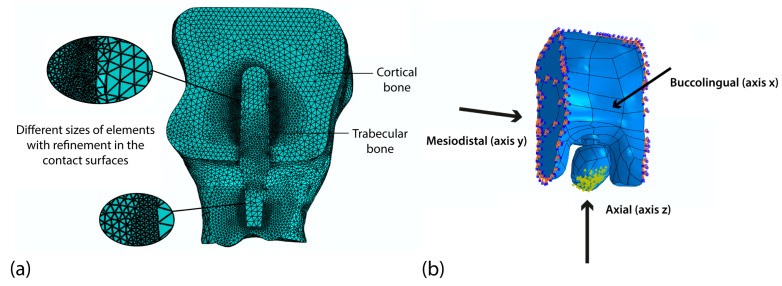
The anchorage of the dental implant in the maxilla with the system mesh (**a**) and boundary conditions and the direction of the loads used in the simulations (**b**).

Interactions were established between the contact surfaces of the system components, considering the physical unions that exist between these elements, with the use of a Tie type restriction, which implies that the condition of the defined surfaces is a single one. A global mesh (Figure 2a), with tetrahedral elements with an approximate size of 0.7 mm was implemented, whereas a locally refined mesh with a size of 0.2 mm was used on contact surfaces. The number of nodes and system elements in all simulations depends on the size of the mesh and varies according to changes in implant geometry. The system with the smallest implant dimensions (3.7 and 8 mm) has the lowest number of elements and nodes (1,826,206 elements and 339,497 nodes), whereas the system with the largest implant dimensions (4.0 and 12 mm) exhibits the highest values for these parameters (1,888,761 elements and 349,037 nodes). To guarantee the accuracy of the stress and strain values obtained, a convergence test was carried out, maintaining the load and boundary conditions. In this test, a tetrahedral mesh with a refinement of 0.16 mm was used for the contact surfaces, and a refinement of 0.6 mm was used in the global mesh. The result was an error of less than 2%.

A delayed loading condition was simulated, considering the implant as fully osseointegrated. Multidirectional occlusal loads were applied simultaneously along three anatomical axes: axial (117 N), bucco-lingual (21.58 N), and mesio-distal (29.48 N). Considering that the implant was anchored at the second premolar site, the applied loads slightly increased compared to those used by Himmlová et al. for a first premolar [62]. The occlusal surface was modeled as the entire crown surface of the premolar, with loads applied across a set of 30 nodes distributed on most of its geometry to ensure physiologically representative force application (Figure 2b). This approach allowed for balanced load transmission while accounting for anatomical variability (e.g., cusp inclination and marginal ridges) to ensure proper load distribution. By considering the entire occlusal surface rather than isolated contact points, the model more accurately replicates in vivo loading conditions, where mastication forces are distributed across the crown. Boundary conditions were implemented by fully constraining the bone model, restricting all degrees of freedom to simulate embedded conditions.

### 2.4. Experimental Design and Statistical Analysis

This study evaluated the influence of two key parameters on the stress and strain levels in peri-implant cortical and trabecular bone. Three different threaded portion lengths (Ls) and two implant neck diameters (Ds) were analyzed using the finite element method simulations. Consequently, the experimental design consisted of six test configurations, as detailed in Table 1. The diameter and length parameter values used in the simulated dental implant models fall within the ranges used in implants produced by various commercial manufacturers. However, for the length values, experimental runs A and D are considered short implants. Six experimental runs were performed using the Ti6Al4V alloy, supplemented by two additional runs (smallest and largest implant designs A and F) employing β-titanium Ti35Nb7Zr5Ta.

All stress and strain values were extracted from the experimental runs in the trabecular and cortical bones, and the 300 most loaded nodes generated by each experimental run were selected to evaluate the biomechanics of the dental implant. The selected nodes were compiled in Microsoft Office Excel; subsequently, the data sheets were exported to StatGraphics Centurion XIX software (v.19) (Statpoint Technologies Inc., Warrenton, VA, USA). In this software, box-and-whisker analysis was used to determine the VMES and aberrant VMS values. The values were subjected to a normality test (Kolmogorov–Smirnov test) and subsequently analyzed using an analysis of variance (ANOVA). Additionally, a Kruskal–Wallis test was used to identify differences between groups, and a value of *p* < 0.05 considered statistically significant.

## 3. Results and Discussion

### 3.1. Stress and Strain Distribution Patterns in Cortical Bone Using Ti6Al4V (α+β Ti)

**Figure 3:**
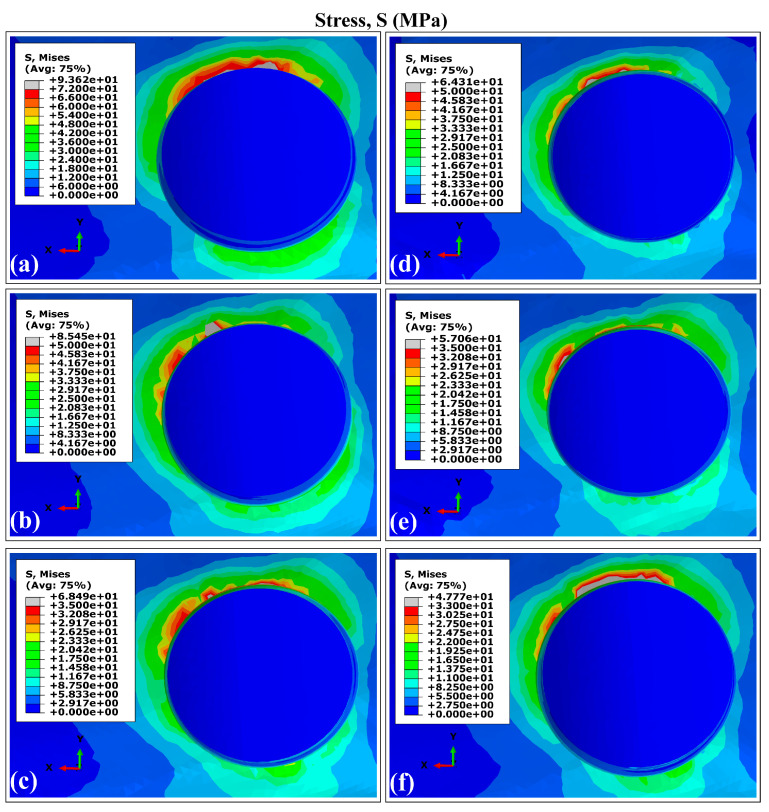
The distribution of von Mises equivalent stresses in the external surface of cortical bone surrounding the Ti6Al4V (α+β Ti) dental implant. Note: The letters indicate the experimental run (see Table 1).

**Figure 4:**
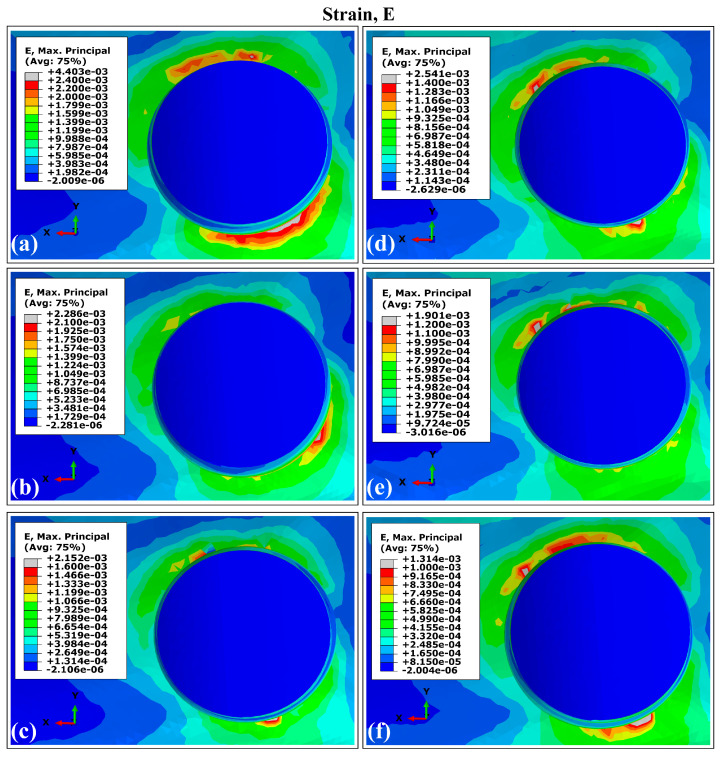
The von Mises strain distribution in the external surface of cortical bone adjacent to the Ti6Al4V (α + β Ti) dental implant. The letters indicate the experimental run (see Table 1).

The VMES distributions and VMS distributions generated in cortical bone by the experimental runs are presented in Figure 3 and Figure 4. All evaluated models produced similar distribution patterns for both parameters, characterized by peak concentrations in peri-implant bone, particularly in the maxillary superior region adjacent to the implant necks. This observed behavior aligns with the findings reported in previous studies involving a finite element analysis of dental implants [40,63,64]. The maximum values for both mechanical parameters were consistently located around the distal aspect, while the minimum values occurred predominantly in areas interfacing with trabecular bone. Regarding the superior maxillary region, the peak values of von Mises equivalent stresses (MVMES) and strains (MVMS) demonstrated dimensional dependence, which varied according to implant geometric parameters.

#### Stress and Strain Distribution Patterns in Cortical Bone Using Ti35Nb7Zr5Ta (β-Ti)

**Figure 5:**
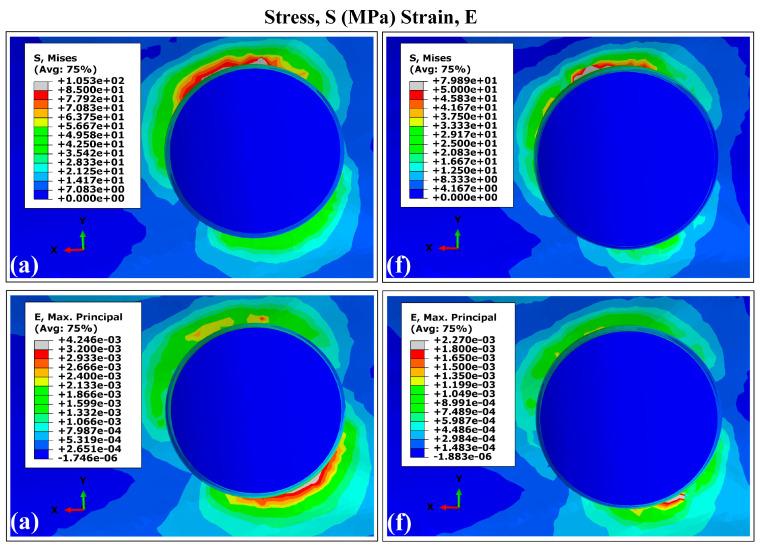
The distribution of von Mises equivalent stresses (S) and von Mises strains (E) in the external surface of peri-implant cortical bone using the Ti35Nb7Zr5Ta (β-Ti) implant. The letters indicate the experimental run (see Table 1).

Figure 5 shows the von Mises stress and strain distributions in cortical bone for implants with smaller and larger dimensions (runs A and F). Both implant designs produced similar stress and strain patterns under the same conditions, showing similar results to those generated by Ti6Al4V dental implants and presenting the highest values concentrated in the peri-implant bone, around the implant neck in the superior maxilla. These results are consistent with previous finite element studies of dental implants [40,59,60]. The maximum values of VMES and VMS were observed at the distal site, while the lowest values were found in regions adjacent to trabecular bone. In the superior maxillary area, the peak magnitudes of VMES (MVMES) and VMS (MVMS) exhibited dimensional dependence, correlating with variations in implant geometry.

### 3.2. Distribution Patterns of Stresses and Strains in Trabecular Bone Using Ti6Al4V (α+β Ti)

**Figure 6:**
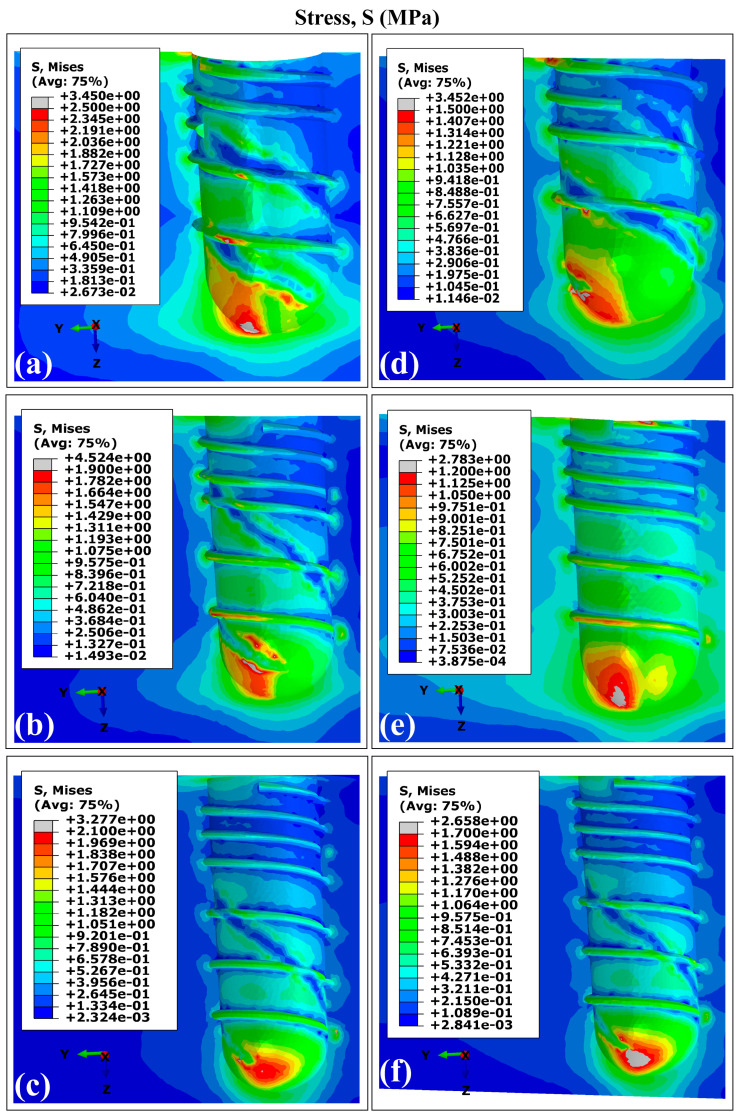
The distribution of von Mises equivalent stresses in the trabecular bone using Ti6Al4V (α+β Ti). The letters indicate the experimental run (see Table 1).

**Figure 7:**
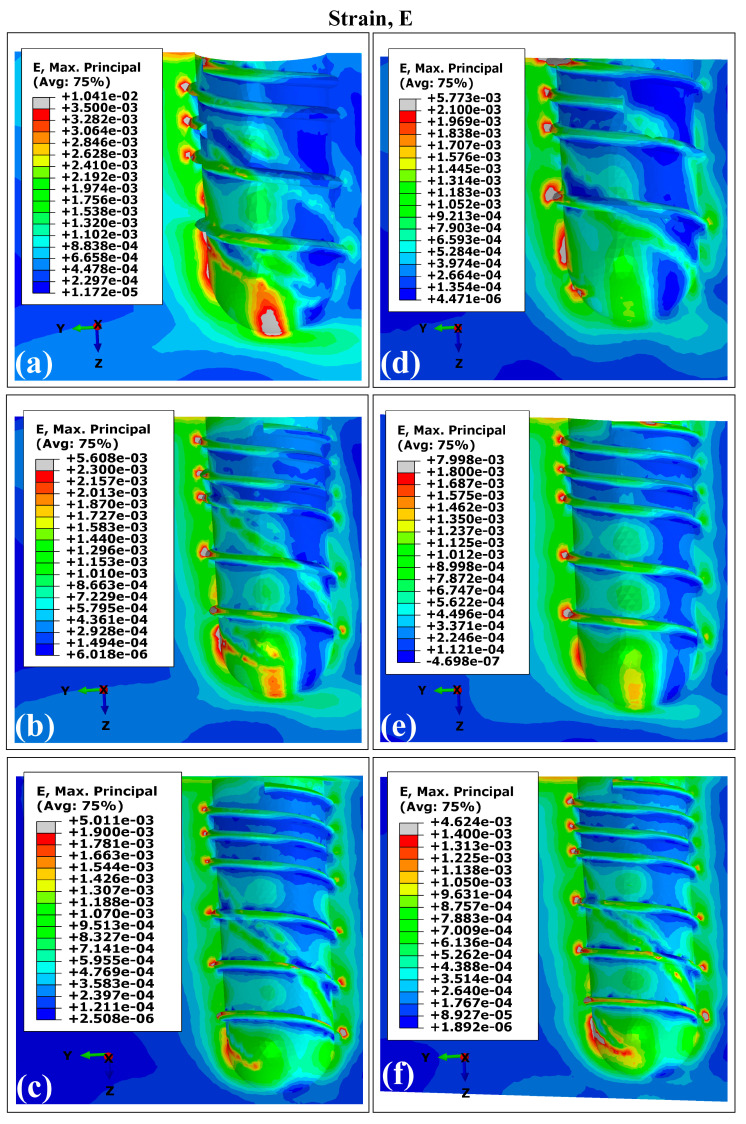
The von Mises strain distribution in trabecular bone using Ti6Al4V (α+β Ti). The letters indicate the experimental run (see Table 1).

Figure 6 and Figure 7 present the VMES and VMS distributions in the trabecular bone for all experimental configurations. The peak values for both parameters were concentrated primarily in two regions: (1) in the interface with the apical zone of the implant, and (2) for strain distributions specifically, the threaded implant surface at the distal site. This mechanical behavior stems from the geometry of the thread acting as a stress concentration feature due to its complex three-dimensional morphology.

The implant models produced comparable stress and strain distribution patterns in the trabecular bone, with reduced values for both mechanical parameters observed in regions interfacing with the double-threaded portion. However, variants C and F (which feature longer stem lengths) demonstrated more uniform distribution patterns. Specifically, these configurations showed diminished differences between VMES and VMS values when comparing distal versus mesial sites, as well as between peri-implant bone regions adjacent to apical versus proximal implant sections.

#### Distribution Patterns of Stresses and Strains in Trabecular Bone Using Ti35Nb7Zr5Ta (β-Ti)

**Figure 8:**
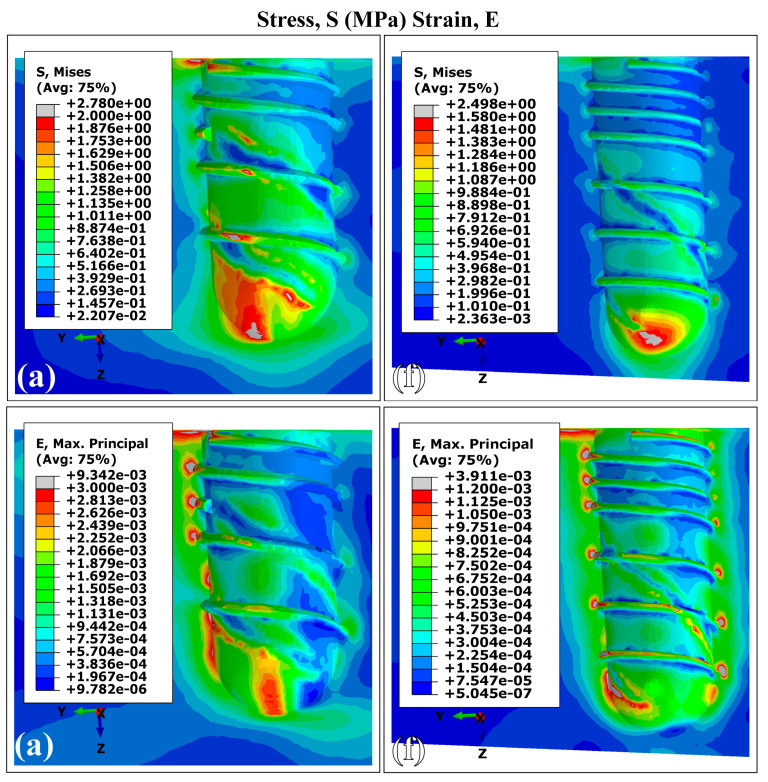
The distribution of von Mises equivalent stresses (S) and von Mises equivalent strains (E) in trabecular bone using Ti35Nb7Zr5Ta (β-Ti). The letters indicate the experimental run (see Table 1).

The FEA of the VMES and VMS distributions in trabecular bone for the largest and smallest implant dimensions fabricated using Ti35Nb7Zr5Ta (β-Ti) are shown in Figure 8. Consistent with the simulations using the Ti6Al4V alloy, the implant with the largest length and diameter dimensions (F) exhibited a significant reduction in the peak VMES and VMS values in the trabecular bone compared to those generated by variant A. Higher VMES values were found at the bone–implant interface near the apical region, while peak VMS values were concentrated around the distal threads, likely due to the abrupt changes in geometry. These results highlight the combined mechanical effect of implant features: the apical engagement supports primary stability, while the thread design affects how strain is distributed locally. Compared to Ti6Al4V, the β-Ti alloy led to a more even distribution of stress along the implant, especially in the apical region, suggesting improved biomechanical performance.

### 3.3. The Influence of the Diameter and Length of the Dental Implants on the Values of MVMES and MVMS in the Peri-Implant Bone

Long-term peri-implant bone quality has been shown to correlate directly with the stress and strain values induced in bone tissue [14,55,65]. Excessive and insufficient mechanical stimulation can adversely affect implant biomechanical performance [66,67]. Typically, the stress peak occurs primarily in peri-implant cortical bone. The underload zones appear in the trabecular bone regions. This stress reduction phenomenon results from the modulus mismatch between implant materials (generally exhibiting high Young’s modulus) and surrounding bone tissue [68,69,70].

In the peri-implant cortical bone, areas with stress values that constitute overloads were observed. However, stress and strain values prevailed within the range that favors bone remodeling and in which bone tissue density is maintained. It is known that overloads can rapidly generate both dimensional losses in the jaws and a decrease in the density of the peri-implant bone [65]. On the other hand, the lower stress values observed in the peri-implant trabecular bone were within the levels considered as underload for quality II bone tissue; this behavior must be related to the stress shielding effect. In the case of the strains, values were lower than 0.003, although in some areas, their levels were close to the recommended lower limit, which could generate a loss of peri-implant bone density [71]. In the cortical and trabecular bones, stress and strain distributions with underload areas were obtained in the peri-implant bone (Figure 3, Figure 4, Figure 6 and Figure 7). Although slower and more moderate than overload, it can affect bone density because insufficient mechanical stimuli sustained over time cause bone tissue to be unable to perform the bone remodeling process and therefore maintain its density [30].

In cortical bone, stress levels were obtained within a range of 30–78 MPa and strains between 0.0010 and 0.0027. These findings partially align with previous studies that analyzed the effect of implant geometry and dimensions on stress levels. Baggi et al. reported, for Nobel Biocare designs, higher stresses (40–90 MPa) in conical implants, suggesting that cylindrical designs, such as the one used in our study, can promote a more favorable load transmission [72]. Meanwhile, Ding et al. observed that increasing implant diameter led to a reduction in bone strains, which is consistent with our findings, although their study evaluated conical implants [73]. In contrast, Muangsisied et al. found that implant length had a limited impact on cortical stresses, whereas our study detected significant variations when modifying this parameter [74]. These discrepancies could be attributed to differences in geometric configurations (cylindrical versus conical) and loading conditions, reinforcing the importance of optimizing implant design based on bone characteristics.

The results obtained in trabecular bone (stresses: 1–3 MPa; strains: 0.002–0.0045) demonstrate certain advantages compared to the previous literature. Although Premnath et al. reported higher stresses (3–6 MPa) in conical implants of similar length, which poses a risk of overloading, most of our stress values remained within a safe range that avoids pathological overload and underload [75]. Particularly relevant is the comparison with Liao et al., who observed excessively low strains (<0.0015) that can lead to bone loss due to insufficient stimulation. In contrast, most of our strain values (0.002–0.0045) fall within the optimal range described by Frost for promoting bone remodeling [71,76]. Furthermore, although Yuan et al. obtained comparable strains (0.002–0.004) in short implants, their stress distribution was less uniform, highlighting how our design optimizes load transfer [77].

The simulation results confirmed previous reports on the influence of dental implant dimensions on biomechanical behavior [62,68]. Experimental configurations combining larger diameters with greater implant lengths (runs E and F in Figure 3, Figure 4, Figure 6 and Figure 7) produced reduced values of the MVMES and the MVMS in both cortical and trabecular bone. In contrast, the highest levels of MVMES and MVMS levels occurred in configurations with smaller diameters and shorter implant lengths (runs A and B in Figure 3, Figure 4, Figure 6 and Figure 7).

**Figure 9:**
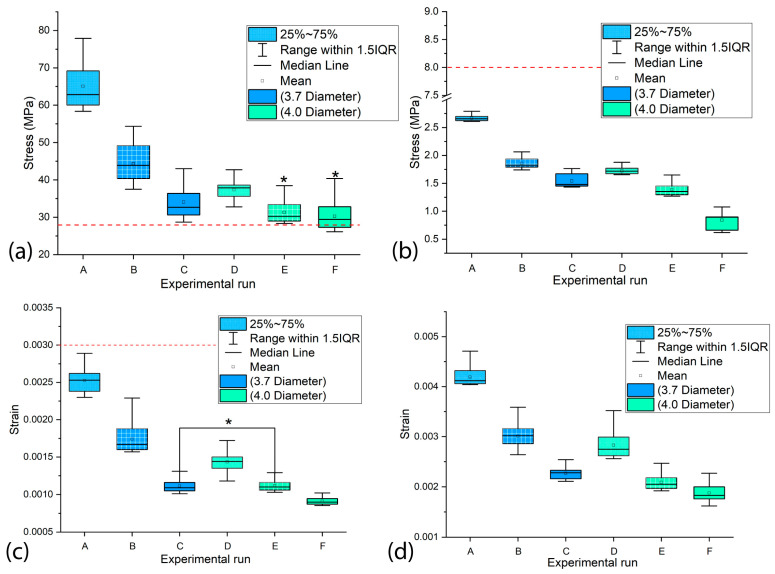
The range of stresses and strains in the most loaded areas of the peri-implant bone using Ti6Al4V (α+β Ti). (**a**,**c**) In the cortical bone; (**b**,**d**) in the trabecular bone. Note: The asterisks indicate that there are no statistically significant differences between the experimental runs, and the red line represents the recommended upper limit.

The analysis revealed that MVMES values in cortical bone and MVMS values in trabecular bone for smaller-diameter implant variants frequently exceeded the thresholds recommended by Li et al. for maintaining peri-implant bone density [78]. Regarding MVMS values, most of the measurements fell within Frost’s proposed range for preventing micro-damage [71]. In general, the VMS values obtained in peri-implant bone remained within safe ranges that avoid micro-damage (Figure 4, Figure 7 and Figure 9), potentially contributing to clinical success. However, experimental variant A in the trabecular bone produced localized strain values marginally exceeding the recommended limits. Such excessive strains (>0.003) may disrupt bone remodeling equilibrium, as established in previous research [71].

Both implant diameter and length demonstrated statistically significant effects (*p* < 0.05) on the MVMES and MVMS values in peri-implant cortical and trabecular bone. Among these factors, implant diameter exhibited the strongest influence on the four response variables, consistent with findings reported in previous studies [62,68]. This phenomenon can be attributed to the biomechanical principle that an increase in the diameter results in a greater stress transfer area at the implant–bone interface. The expanded load-transfer surface promotes more effective stress dissipation, consequently reducing stress and strain magnitudes in peri-implant bone.

The biomechanical performance of dental implants was further assessed by analyzing the stress and strain ranges in the most heavily loaded peri-implant bone regions (Figure 9). Figure 9a,c show the maximum VMES and VMS ranges in cortical bone that experience peak loading. Statistical analysis revealed significant differences (*p* < 0.05) in both stress and strain values among most experimental configurations, with the exception of variants E–F (VMES comparison) and C–E (VMS comparison), which did not show statistically significant variations. In trabecular bone, all experimental runs demonstrated statistically significant differences (*p* < 0.05) in the maximum stress and strain ranges, as illustrated in Figure 9b,d.

### 3.4. The Influence of the Type of Material on the Values of MVMES and MVMS in the Peri-Implant Bone

**Figure 10:**
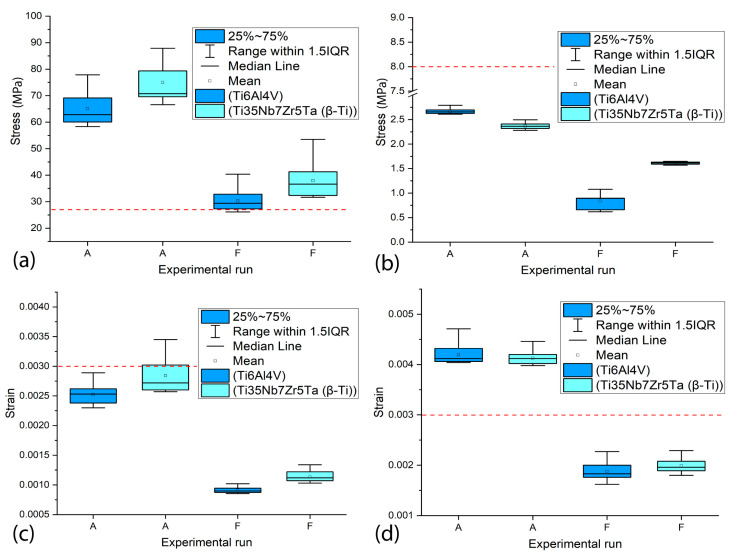
The range of stresses and strains in the most loaded areas of the peri-implant bone for experimental runs with smaller and larger-sized implants (A and F runs). (**a**,**c**) In the cortical bone; (**b**,**d**) in the trabecular bone. Note: The red line is the recommended upper limit.

The influence of the two titanium alloys used in the simulations on the VMES and VMS generated in the peri-implant bone was analyzed. Figure 10 shows the range of VMES and VMS values obtained in the most loaded regions of the cortical bone (a and c) and the trabecular bone (b and d), resulting from experimental runs with smaller and larger-sized implants (A and F). In both regions, all variants exhibited statistically significant differences (*p* < 0.05).

The results demonstrate that the choice of titanium alloy significantly influences the magnitude of stresses and strains transmitted to the cortical bone. The Ti35Nb7Zr5Ta (β-Ti) alloy, characterized by a lower elastic modulus compared to Ti6Al4V, consistently generated higher stresses (90 MPa vs. 78 MPa in smaller-sized implants; 40 MPa vs. 30 MPa in larger-sized implants). This difference can be attributed to its greater flexibility, which reduces the overall stiffness of the implant–bone system, slightly increasing the load transfer to the adjacent bone tissue. However, this behavior does not necessarily represent a disadvantage as moderate mechanical stimulation could promote bone remodeling, provided that damage thresholds are not exceeded. Regarding strains, although β-Ti yielded slightly higher values (0.0030 vs. 0.0025 in smaller implants; 0.0012 vs. 0.0010 in larger implants), they never exceeded the physiological limit (0.003), suggesting that both alloys are mechanically biocompatible. Nevertheless, the lower stiffness of β-Ti could be advantageous in clinical scenarios aiming to mitigate stress-shielding effects, particularly in patients with low bone density.

In the trabecular bone, significant differences in biomechanical response were observed depending on the alloy used. For the smaller-sized implant, Ti6Al4V generated slightly higher stresses (2.7 MPa) compared to β-Ti (2.5 MPa), while in the larger-sized implant, the same behavior observed in cortical bone was maintained, with β-Ti showing higher values (1.7 MPa vs. 1 MPa for Ti6Al4V). Regarding strains, in the smaller implant experimental variants, similar values were obtained for both alloys (0.0044 for Ti6Al4V and 0.0042 for β-Ti) that slightly exceeded the physiological limits for maintaining bone density (0.003), which could potentially lead to bone loss due to mechanical overload. In contrast, the larger-sized implants maintained strains within the safe range (0.002–0.0022), although β-Ti again showed higher values.

The results demonstrate that the choice of titanium alloy significantly influences the biomechanical response of both cortical and trabecular bone. Due to its lower elastic modulus, the Ti35Nb7Zr5Ta alloy (β-Ti) generated different stress and strain values compared to Ti6Al4V, with a tendency to transfer higher loads to bone tissue. In cortical bone, this behavior remained within physiological limits for strain values, suggesting that β-Ti may reduce the risk of stress shielding. However, in trabecular bone, both materials induced critical strains in short implants, although β-Ti demonstrated better performance in terms of stress distribution. These findings highlight that while β-Ti offers theoretical advantages for bone remodeling, its clinical application requires careful consideration of implant design and patient bone quality to prevent harmful overloads, particularly in trabecular bone regions.

For the clinical implementation of the developing implant model, evidence suggests preferential selection of implant variants of maximum diameter and maximum length, contingent on adequate dimensions of the recipient jawbone. However, several study limitations constrain the direct clinical extrapolation of these findings. First, the simulation methodology used static loading conditions under delayed loading assumptions. Second, critical variables including peri-implant bone quality variations and implant positioning within the jaw were not incorporated in the biomechanical analysis. Consequently, future research should focus on (1) using FEM to investigate the effects of these unexamined variables on peri-implant stress/strain distributions and (2) validating current findings through experimental testing, including controlled in vivo experimentation.

## 4. Conclusions

This investigation employed finite element analysis to assess the biomechanical performance of a novel dental implant design, with particular focus on evaluating how the implant length and diameter influence MVMES and MVMS in peri-implant bone. The main findings are as follows:

1. Peak stress concentrations occurred in peri-implant cortical bone, particularly in the maxillary surface region adjacent to the implant neck. This mechanical behavior stems from the elevated Young’s modulus of the cortical bone, which enhances its load-bearing capacity. On the contrary, the maximum strain values were localized in the trabecular bone regions.
2. Both the implant diameter and length demonstrated statistically significant effects (*p* < 0.05) on the peak equivalent stress and strain values in cortical and trabecular bone.
3. Implant diameter emerged as the dominant variable affecting mechanical response, attributable to increased implant–bone contact area that promotes load dissipation and consequently reduces peri-implant stress/strain magnitudes.
4. Superior biomechanical outcomes consistently correlated with larger implant dimensions, suggesting enhanced clinical performance potential for crown–implant systems featuring maximum diameter and length configurations.
5. The material of the dental implant demonstrated a statistically significant influence (*p* < 0.05) on the maximum levels of stress and strain generated in the cortical and trabecular bones. In general, the Ti35Nb7Zr5Ta alloy implants showed higher maximum values of VMES and VMS than those generated by Ti6Al4V alloy implants.

## Figures and Tables

**Table 1:** Experimental design.

Experimental Run	Implant Parameter	
	D (mm)	L (mm)
A	3.7	8
B	3.7	10
C	3.7	12
D	4.0	8
E	4.0	10
F	4.0	12

**Table 2:** The properties of the materials used in the system components.

Material	Young’s Modulus, E (MPa)	Shear Modulus, G (MPa)	Poisson’s Ratio, ν	References
Ti6Al4V (dental implant)	110,000	-	0.32	[58]
Ti35Nb7Zr5Ta (β-Ti) (dental implant)	55,000	-	0.32	[59]
Feldspathic ceramic (crown)	82,800	-	0.35	[60]
Cortical bone	Ex = 17,900 * Ey = 26,600 Ez = 12,500	Gyx = 4500 Gyz = 7100 Gxz= 5300	νxy = 0.26 νxz = 0.31 νyz = 0.28	[61]
Trabecular bone	Ex = 1148 Ey = 1148 Ez = 21	Gyx = 68 Gyz = 434 Gxz = 68	νxy = 0.05 νxz = 0.055 νyz = 0.322	

* X—bucco-lingual direction; Y—mesio-distal direction; Z—axial direction (infero-superior).

## Data Availability

The original contributions presented in this study are included in the article. Further inquiries can be directed to the corresponding authors.

## Abbreviations

The following abbreviations are used in this manuscript:

FEA	Finite element analysis
VMES	von Mises equivalent stress
VMS	von Mises strain
MVMES	Maximum von Mises equivalent stress
MVMS	Maximum von Mises strain
